# Never Out of the Woods: Onset of Events in Long QT Syndrome Late in Life Provoked by Atrial Arrhythmias

**DOI:** 10.1016/s0972-6292(16)30798-7

**Published:** 2014-10-06

**Authors:** Justin Ng, Chirag Barbhaiya, Tobias Reichlin, Koichi Nagashima, Roy John

**Affiliations:** Brigham and Women's Hospital, United States

**Keywords:** Long QT Syndrome, Atrial Arrhythmias

## Abstract

The assessment of risk in the asymptomatic patient with long QT syndrome can often be a challenging task, particularly when the available evidence is limited to relatively small retrospective registries, not to mention the need to consider the effect of individual patient factors which are often difficult to quantitate. We describe the relatively uncommon case of a man with a long-standing diagnosis of Long QT 2 syndrome who suffered his first cardiac event in his late 60's, likely precipitated by the development of paroxysmal atrial tachycardia. A brief review of the available literature on risk assessment in adults with genetically confirmed long QT syndrome who have remained asymptomatic late into adulthood will follow the case.

## Introduction

The assessment of risk in the asymptomatic patient with long QT syndrome can often be a challenging task, particularly when the available evidence is limited to relatively small retrospective registries, not to mention the need to consider the effect of individual patient factors which are often difficult to quantitate. We describe the relatively uncommon case of a man with a long-standing diagnosis of Long QT 2 syndrome who suffered his first cardiac event in his late 60s, likely precipitated by the development of paroxysmal atrial tachycardia. A brief review of the available literature on risk assessment in adults with genetically confirmed long QT syndrome who have remained asymptomatic late into adulthood will follow the case.

## Case Study

A 68 year old male was formally diagnosed with congenital long QT syndrome at the age of 50 and subsequently shown to have an abnormality of the HERG gene confirming the LQT-2 syndrome. ECGs at diagnosis showed sinus rhythm, partial RBBB and QTC of 500 msec with bifid T waves. He was treated with Nadolol 20 mg daily. Three of his sons died at ages 11, 10 and 4 weeks, autopsies in all three showed no evidence of structural abnormalities. His father died suddenly aged 39. The patient himself had remained asymptomatic until a year ago when he experienced a pre-syncopal event with prodrome on waking one night for micturition. Based on the clinical diagnosis and high suspicion for arrhythmias, a loop monitor was implanted.

**Figure 1:**
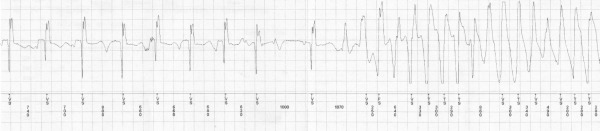

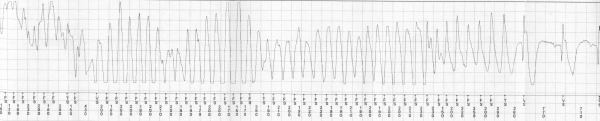
Loop recording of event showing initiation with a short-long sequence created by a blocked PAC (arrow in top panel). The QT interval following the pause is longer and triggers Torsade de Pointes VT that self terminates after 26 seconds (bottom panel).

**Figure 2:**
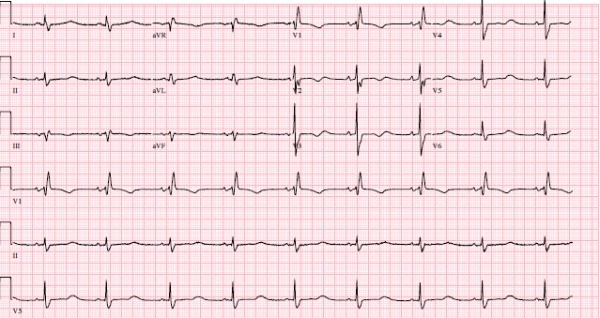
12 lead EKG at presentation showing a prolonged QTC with a RBBB and bifid T waves characteristic of Long QT 2.

**Figure 3:**
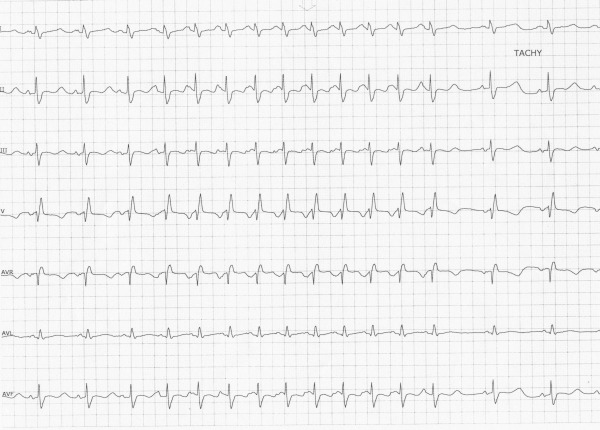
Burst of AT followed by a compensatory pause with prolongation of the QTC.

**Figure 4:**
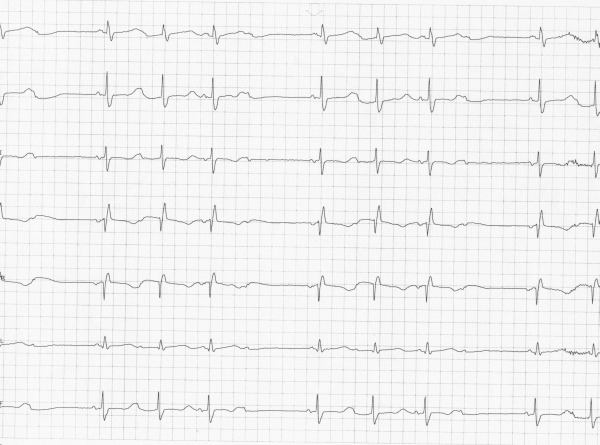
Blocked PACs creating short-long sequences and pause dependent QTC prolongation

On the day of his presentation, he was taking out the garbage when he lost consciousness. There was no warning and the episode was unwitnessed. On regaining consciousness he presented to the emergency department. Interrogation of the implanted loop monitor showed an episode of polymorphic VT (Torsade de Pointes VT) triggered by a short-long sequence (Figure 1) that corresponded temporally to his syncopal event. No other precipitating factors were identified; in particular he reported compliance with his beta-blocker, was not suffering from an inter-current illness and had not commenced any new medications. At the time of presentation, his 12 lead electrocardiogram showed sinus rhythm 54 bpm with a long standing right bundle branch block, PR duration of 154 ms and QRS duration of 128ms. His corrected QT interval was prolonged at 641ms (Figure 2). His electrolyte studies were unremarkable. Whilst on telemetry overnight awaiting ICD implantation, he was noted to have runs of atrial tachycardia followed by a pause and marked prolongation of his QT interval (Figure 3). In addition there were episodes of blocked premature atrial contractions leading to short-long sequences and QT prolongation (Figure 4).

It is our hypothesis that the development of atrial arrhythmias with blocked atrial premature beats due to prolongation of the AV nodal refractory period from beta blockade was the likely precipitant of our patient's event. The patient underwent placement of a dual chamber ICD that was programmed to provide pacing in the DDI mode at 70 bpm with an AV delay of 250 msec to permit native AV conduction. Detection and therapy for ventricular fibrillation was programmed at 220 bpm with a significant delay to detection to prevent therapy for non-sustained VT. Nadolol was increased to 20 mg twice daily. He has remained asymptomatic during a 6 month period of follow up.

## Discussion

Our patient presents a somewhat unusual case of a man with Long QT-2 syndrome who had remained asymptomatic into late adulthood only to have his first cardiac event at the ripe old age of 68. Much of the literature regarding the natural history of long-QT syndrome has been derived from the International Long-QT syndrome registry examining probands and their 1st and second degree relatives [1-5]. The data however, focuses on childhood when most of the events occur [1], and only recently has been extended into early adulthood but still only including patients less than 40 years of age [4]. Certainly there is limited data on patients who have survived into their late 60s without an event, when logically one would expect that the risk of a future event would be relatively low.

From the available literature, it appears that gender and age play a significant role in influencing the course of LQTS [1-4]. Cardiac events tend to occur more frequently in children [1]. The analysis of the International Long-QT Registry data shows that the risk of a cardiac event is higher in males until puberty and then in females thereafter. In fact, a sentinel cardiac event after age 15 occurred in only 8% of males compared to 40% of females [1]. The studies appear to suggest that the rate of increase in cardiac events generally plateaus in adult men however this does not appear to be the case in women particularly with Long-QT2 [2].

Perhaps the most common reason for an event in later life would be the introduction of a QTC prolonging medication. Indeed, there were periods of time in our patient's history where he was closely monitored during the introduction of potentially QTC prolonging antidepressant medications, however this was not a contributing factor to his recent cardiac event. It is our belief that the development of atrial tachycardia and blocked AV nodal conduction to premature atrial beats created the perfect conditions for pause dependent QTC prolongation.

Viskin et al reported on the response of the QT interval to the brief tachycardia provoked by standing identifying an abnormal response in patient's with long QT syndrome and particularly in those with long QT 2 gene [5]. The group found an exaggerated 'stretching' of the QTC in patients with long QT 2 compared with normal controls during changes in heart rate [5]. Such exaggerated responses of repolarization could explain the occurrence of Torsade de Pointes VT in response to atrial arrhythmias in our patient.

## Conclusion

This case provides an important lesson to remain vigilant in assessing risk in patients with genetic syndromes including long QT syndrome, as risk does not remain static over time and can be modified by a number of different environmental factors as well as many conditions which often occur in later life.
